# Epstein-Barr virus and Burkitt’s lymphoma. Associations in Iraqi Kurdistan and twenty-two countries assessed in the International Incidence of Childhood Cancer

**DOI:** 10.1186/s13027-022-00452-0

**Published:** 2022-07-27

**Authors:** Dana N. Muhealdeen, Alan Shwan, Rafil T. Yaqo, Hemin A. Hassan, Bryar O. Muhammed, Rawa M. Ali, Michael D. Hughson

**Affiliations:** 1 Sulaimani University College of Medicine, Sulaymaniyah, Iraq; 2 Hiwa Cancer Hospital, Sulaymaniyah, Iraq; 3 Dohuk University School of Medicine, Dohuk, Iraq; 4 Shorsh Teaching Hospital, Quirga Road, Sulaymaniyah, Iraq

**Keywords:** Epstein-Barr virus, Burkitt's lymphoma, Pediatric lymphoma, Middle East, EBV seropositivity

## Abstract

**Background:**

Burkitt's lymphoma (BL) has worldwide variations in incidence that are related to the age of Epstein-Barr virus (EBV) infection. This study examined the age-specific incidence rate (ASIR) of BL and community EBV seropositivity in Iraqi Kurdistan and compared results with data from countries tabulated in the International Incidence of Childhood Cancer volume 3 (IICC-3).

**Methods:**

The ASIR (95% confidence intervals) of BL in Sulaimani Governorate of Iraqi Kurdistan were calculated for the years 2010–2020. Specimens from 515 outpatients were tested for IgG and IgM antibodies to EBV viral capsid antigen.

**Results:**

In Sulaimani, 84% of BL occurred under 20 years of age, with an ASIR of 6.2 (4.7–7.7) per million children. This ASIR was not significantly different than that of Egypt, Morocco, Israel, Spain, or France. It was slightly higher than the ASIR of the United States, the United Kingdom, and Germany and markedly higher than for Asia and South Africa. In Africa and much of Asia, early childhood EBV exposure predominates, with nearly all children being infected by 5 years of age. In Sulaimani, just over 50% of children were EBV seropositive at 3 years old and 90% seropositivity was reached at 15 years of age. In Europe and North America, seropositivity is commonly delayed until adolescence or young adulthood and adult predominates over childhood BL.

**Conclusion:**

In the Middle East, childhood BL is relatively common and adult BL is rare. In Sulaimani, EBV seropositivity increases progressively throughout childhood and reaches 92% at mid-adolescence. This may reflect the Mid East more widely. We suggest that the high childhood and low adult BL rates may be a regional effect of a pattern of EBV exposure intermediate between early childhood and adolescent and young adult infections.

## Introduction

Burkitt lymphoma (BL) has three recognized subtypes [1, 2]. Most BL is a sporadic disease [3]. The sporadic disease is seen worldwide, with notable geographic differences in overall and age-specific incidence rates (ASIR) [2]. In many parts of the world, sporadic BL is predominantly a childhood disease, but in Europe, North America, and parts of Asia, adult outnumbers childhood BL by more than 2:1 [4, 5]. An endemic type of the disease appears restricted to Equatorial Africa and New Guinea [3, 6]. The endemic BL is the most common specific form of childhood cancer in the affected regions and has a uniformly high age-specific incidence rate [2, 6]. The third subtype involves immunocompromised individuals, most frequently as a complication of HIV infection [3]. Immunocompromise-related BL occurs among children and adults in Sub-Saharan Africa and overlaps with the endemic disease [6, 7].

BL is related to latent Epstein-Barr virus (EBV) infection, with the extent of the relationship varying geographically and with different disease subtypes. EBV-encoded small nuclear non-polyadenylated mRNA (EBER) is found in nearly all African endemic BL but in only 10-40% of sporadic BL in the United States (US) and Europe [2, 3]. In Egypt and Iraq, EBER is demonstrated in more than 70% of tumors [8–11].

EBV exists as a latent infection in more than 90% of the world’s population, but outside of Equatorial Africa, BL is not one of the more common childhood cancers [2]. Because of this disparity, endemic BL is thought to be related to the chronic immune stimulation of EBV-containing lymphoid cells by co-infecting falciparum malaria rather than EBV latency by itself [2, 3].

EBV is a human DNA virus that infects different populations at different ages [12]. In underdeveloped countries, including Africa, EBV infections are asymptomatic or only mildly symptomatic, and 95% or more of the population acquires the virus in early childhood before five years of age [12, 13]. In North America, Western Europe, Japan, and South Korea, EBV exposure is often delayed until adolescence, when it can cause infectious mononucleosis [12–14]. Despite the delayed presentation, the EBV serological prevalence in these countries is greater than 70-80% by mid-adulthood.

In a Qatar study, Smatti et al. [15] examined blood donor samples from Qataris, Jordanians, Palestinians, Saudi Arabians, and Indians and found that more than 95% of persons from all countries were serologically positive by 19 years of age. The age of infection in the Middle and Near East is thought to be early childhood. Yet, studies from Bahrain and Tehran show primary infections in adolescents and young adults that indicate a regional change in the age of EBV exposure may be taking place [16, 17].

In a previous publication, we analyzed rates of lymphoma in Iraqi Kurdistan over the years 2010-2014 and suggested that our BL might be four times as frequent as it is in children of similar age in the United States (US) [9]. BL in the Middle East may have elements of an endemic disease; nevertheless, no coexisting infection or promoting factor other than EBV has ever been identified [8–10].

To further evaluate the regional frequency of BL and its relationship to EBV, we estimated the ASIR in the Sulaimani Governorate of Iraqi Kurdistan over an 11 year period and performed EBV serological tests on 515 non-cancer outpatients. The findings were compared with data from countries tabulated in the World Health Organization's International Incidence of Childhood Cancer Volume 3 (IICC-3) [18] to investigate how local BL incidence rates and ages of EBV exposure correspond to other regions of the world.

## Methods

### Database and calculation of incidence rates

Hiwa Hospital was established in 2005 for public cancer care in the Sulaimani Governorate and to develop a population-based cancer registry. The Pathology Department of Shorsh Teaching Hospital provides surgical pathology services and central pathology reviews for Hiwa patients. Clinical and pathology reports on BL from Hiwa Hospital and the Shorsh Pathology Department were collated for the period January 1, 2010 to December 31, 2020. Pediatric data for Egypt, Algeria, Morocco, Jordan, Israel, Saudi Arabia, Turkey, South Arica, Uganda, Cameroon, the US, Canada, the United Kingdom (UK), Germany, France, Italy, Spain, Japan, Korea, China, Thailand, Korea, and India were obtained for BL from IICC-3 tables [17]. Adult data and EBV relationships were obtained from individual articles from these countries [4, 7, 8, 15, 19–42].

Pediatric patients were defined as 0-19 years of age. For Sulaimani, BL ASIR was calculated as the annual average number of cases. The population of Sulaimani at 2,095,851 was obtained from the 2015 Iraqi Cancer registry. The 0-19-year-old population was 910,510. Age-adjusted incidence rates (AAIR) were estimated using the 2001 WHO World Standard Population [43].

### Diagnostic criteria

**Fig. 1:**
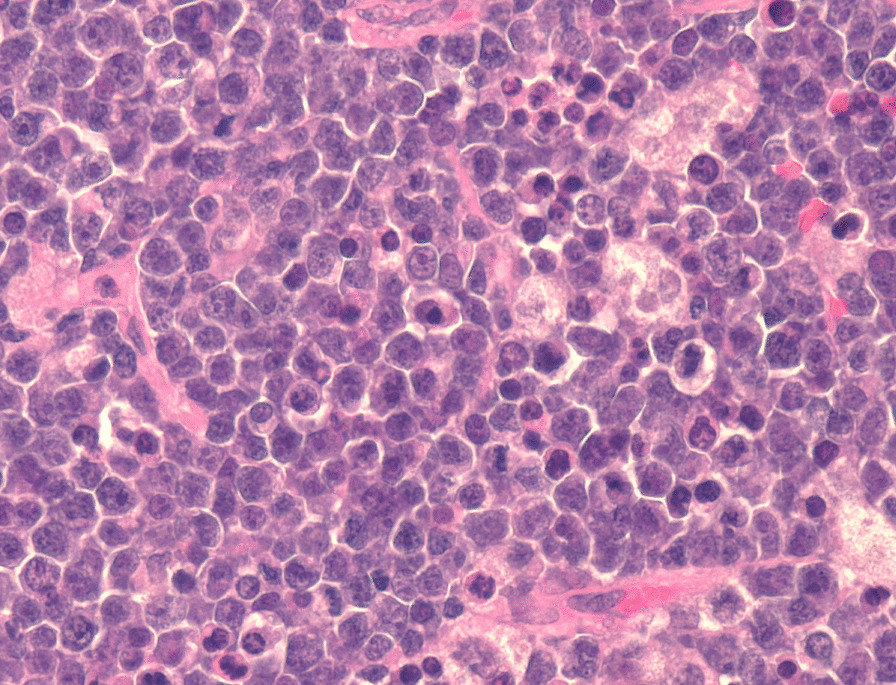
Hematoxylin and Eosin 400X. Burkitts lymphoma in a cervical lymph node of a two-year-old girl. The girl was serologically negative for Epstein-Barr virus infection. The tumor was positive for the MYC translocation but negative for Epstein-Barr virus encoded small nuclear mRNA

BL was diagnosed by flow cytometry on fine needle aspirations (FNA) or by immunohistochemistry on formalin-fixed, paraffin-embedded tissue sections. Histology on tissue sections or FNA cell blocks showed rapidly proliferating, undifferentiated lymphoid cells having abundant blue cytoplasm with cytoplasmic vacuoles [Fig. 1]. All tumors contained tingible body macrophages. BL was diagnosed when cells were CD20, CD10, and BCL6 positive with bright light chain restricted surface immunoglobulin and when the Ki67 proliferation index approached 100% [2]. TdT was required to be negative. Weak or focal BCL2 was acceptable when other markers were characteristically positive.

### EBV latency and MYC translocations in BL

BL EBV latency was assessed by chromogenic in situ hybridization (ISH) using the Novocastra (Leica Biosystems, Wetzler, Germany) probe for EBER. C-MYC/IGH translocations were analyzed by fluorescence in situ hybridization using a break-apart probe (Vysis MYC, Abbott Laboratories, Abbott Park, IL). EBER and MYC translocation studies used formalin-fixed, paraffin-embedded tissue of 24 BL.

### EBV serological testing

Samples in serum separator tubes were obtained from 515 patients attending general pediatric and medical clinics from March 13-31, 2022. Specimens were used for serum chemistries and held at 4 °C for 48 hours. The specimens were de-identified for all information except age. The samples would otherwise have been discarded, and the Directorate of Health allowed their use.

Samples were analyzed by patient age for IgG and IgM antibodies against EBV viral capsid antigen (VCA) using the Abbot Architect model i1000 immunoassay system (Abbot Diagnostics, Wiesbaden, German). The results were instrument recorded as negative or positive, with positive but not negative results additionally provided as relative light units.

### Data analysis

Data were entered into Excel worksheets and analyzed with Excel mathematical functions and Stata statistical software version 10.0 (StataCorp, College Station, TX). Uncertainty for ASIR was evaluated by comparing 95% confidence intervals in the different geographical regions. The calculation of ASIR 95% CI used the following formula:95% CI (ASIR) = ±1.96 X √(Ri^2^/Ni) where Ri^2^ = age-specific incidence in the specified age group, and Ni = the number of patients in the age-specific group [44]. Differences were considered significant if 95% CI did not overlap.

## Results

### BL in Sulaimani, general characteristics and incidence

In the 2010-2020 period, 74 cases of BL were diagnosed in the Sulaimani Governorate. The characteristics of the patients are shown in Table 1.

**Table 1:** Characteristics of patients diagnosed with Burkitt lymphoma in the years 2010 through 2020 in Iraqi Kurdistan

Characteristics	Burkitt lymphomas
No. of patients	74
Male, no. (%)	65 (88%)
*Age, yrs: range, median (IQR)*	1.5–56, 5.5 (4–12)
0–4, no. (%)	28 (38%)
5–9, no. (%)	22 (30%)
10–14, no. (%)	10 (14%)
15–19, no. (%)	2 (3%)
≥ 20, no. (%)	12 (16%)
Immunosuppressed	1 (HIV, hemophilia)
*Presenting site, no. (%)*	
Abdomen/intestine	52 (70%)
Cervical LN	10 (14%)
Tonsil	4 (5%)
Mediastinum	1 (1%)
Kidney	2 (3%)
Breast	1 (1%)
No information	2 (3%)

*IQR* interquartile range; *LN* lymph node.

The BL patients were 88% male, 84% were 0-19 years old, and 38% were 0-4 years old. Only one patient was immunosuppressed, a 25-year-old HIV-positive hemophiliac male. In 70% of patients, the presentation was an abdominal tumor. Cervical lymph node enlargement was next at 14%. Two patients had soft tissue facial masses, but neither had any facial bone involvement. A 32-year-old woman presented with bilateral breast tumors. Two males, four and five years old, had primary kidney tumors. The abdominal tumors were diagnosed by FNA and flow cytometry in 37 patients and by core or incisional biopsies in 17 patients. All diagnoses in non-abdominal sites were established by biopsies.

Twenty-four tumors were tested for EBER and the MYC translocation, all from patients under 20 years old. The blocks were obtained from the first 40 patients with biopsies (not FNA cell blocks) obtained in 2012-2018 and were selected only for the adequacy of tissue. The tissue was from kidney, 2 cases; tonsil, 2 cases; cervical lymph, 8 cases; and abdominal tumor, 12 cases. EBER was positive in 20 cases (83%), and all 24 tumors (100%) were positive for the MYC translocation. The EBER negative cases were cervical lymph node, 2 cases, and abdominal tumor, 2 cases. One two-year-old patient with an EBER negative cervical lymph node was serologically EBV negative at the time of biopsy (Fig. 1). This was the only patient on which serological status was known. The other EBER negative cases were 4, 7, and 11 years old.

The ASIR (95% CI) for BL in Sulaimani was 6.2 (4.7-7.7) per million children 0-19 years old. Only 3% were 15-19 years old, with 97% being nearly equally divided between the ages of 0-4 and 5-14.

### Incidence of pediatric BL worldwide

Table 2 lists by country the ASIR (95% CI) of pediatric BL, the frequency of males, and the proportion of patients in the 0-5-, 5-14-, and 15–19-year-old age ranges. The table provides the survey period and the number of patients in the IICC-3 registries [18].

**Table 2:** Burkitt’s lymphoma. The table lists the age-specific incidence rate per million children and the distribution of patients in the 0–4-, 5–14-, and 15–19-year-old age ranges. ]

Country	Years data collected	Cases no.	ASIR (95% CI) per million children, 0–19 yrs old	Male	Age distribution, years		
					0–4	5–14	15–19
Sulaimani	2010–20	62	6.2 (4.7–7.7)	0.89	0.45	0.52	0.03
Egypt	1996–2010	97	5.2 (4.2–6.2)	0.73	0.47	0.46	0.07
Algeria	1996–2014	23	1.0 (0.6–1.4)	0.81	0.43	0.41	0.15
Morocco	2005–2012	60	5.0 (3.7–6.3)	0.82	0.24	0.62	0.14
Jordan	2000–2012	129	3.7 (3.1–4.3)	0.78	0.27	0.64	0.09
Israel-Jew	1990–2012	195	5.0 (4.3–5.7)	0.74	0.32	0.52	0.15
Israel-non Jew	1990–2012	69	4.8 (3.7–5.9)	0.77	0.23	0.63	0.14
Saudi Arabia	1994–2012	121	2.8 (2.3–3.3)	0.68	0.35	0.53	0.13
Turkey	1992–2012	180	3.5 (3.0–4.0)	0.70	0.28	0.64	0.08
S. Africa-black	1998–2012	252	1.4 (1.2–1.6)	0.75	0.50	0.50	Na
S Africa-white	1998–2012	34	2.8 (1.9–3.7)	0.75	0.19	0.81	Na
Uganda	1996–2013	376	21.6 (19.4–23.8)	0.56	0.20	0.76	0.04
Cameroon	2004–2006	122	40.0 (32.9–47.1)	0.58	0.23	0.64	0.07
Canada	1992–2013	408	2.5 (2.3–2.7)	0.80	0.18	0.59	0.22
US-black	1998–2012	276	1.4 (1.2–1.6)	0.74	0.17	0.63	0.20
US-white	1998–2012	2072	2.8 (2.7–2.9)	0.80	0.16	0.64	0.23
UK	2000–2011	419	2.3 (2.1–2.5)	0.79	0.21	0.62	0.17
Germany-West	1994–2012	198	2.8 (2.4–3.2)	0.82	0.14	0.67	0.18
France	1993–2012	182	4.3 (3.7–4.9)	0.81	0.19	0.68	0.19
Italy	1992–2013	224	4.0 (3.5–4.5)	0.76	0.14	0.68	0.24
Spain	1990–2013	210	4.8 (4.2–5.5)	0.81	0.25	0.59	0.19
Japan	1990–2013	110	1.4 (1.1–1.7)	0.85	0.26	0.66	0.08
Korea	1999–2012	351	2.1 (1.9–2.3)	0.80	0.28	0.59	0.13
China	1990–2013	33	0.4 (0.3–0.5)	0.78	0.33	0.61	0.06
Thailand	1993–2013	41	0.8 (0.6–1.0)	0.81	0.42	0.45	0.13
India	1990–2013	109	0.5 (0.4–0.6)	0.70	0.32	0.63	0.05

Data from Sulaimani is local. Data from the other countries are compiled from the tables of diagnostic subgroups in the WHO IICC-3 website [https://iirc.iarc.fr/results/comparative.php

*ASIR* age-specific incidence rate, *95% CI* 95% confidence interval, *na* information not available.

The ASIR in countries close to each other were similar. The Equatorial African nations of Uganda and Cameroon harbor endemic forms of BL and have 0–19-year-old ASIRs of 21.6 in Uganda and 40.0 in Cameroon. In South Africa, rates of BL are much lower than in Uganda and Cameroon, and South African blacks have significantly lower rates than South African whites. This racial difference is also seen in the US, where rates of BL among blacks are half that of whites. Also, note that the recorded US and South African pediatric rates are the same for blacks and whites.

The countries of the Middle East have ASIRs ranging from 2.8 to 6.2 that resemble the rates in France (4.3), Italy (4.0), and Spain (4.8). In Israel, the ASIR is essentially the same between Jews (5.0) and non-jews (4.8). Saudi Arabia with an ASIR of 2.8 is low for the region, but the 95% CI overlaps with the rates in Turkey and Jordan, and the differences in ASIR between these three countries is not significantly different. Algeria, of North Africa, has an ASIR of 1 per million children that is significantly lower than nearby Morocco (5.0) to the west and Egypt (5.2) to the east. The Far East and India have very low rates of BL (< one child per million). The populous six regions registry in China with an ASIR of 0.4 recorded only 33 pediatric BL in the 23-year period, 1990-2013.

In all parts of the world, BL is a predominantly male disease, with the endemic BL of Equatorial Africa having somewhat less gender disparity than other regions. In all regions, 78% to more than 95% of pediatric patients have symptomatic tumors by 14 years of age. However, it is notable that Europe, the US, and Canada occupy the low end of this range, and in these countries, more than 16% of pediatric BL is found at 15-19 years old.

### *Pediatric versus adult BL and the relationship to the age of EBV infection (Table **3**)*

**Table 3:** Comparison of regional Burkitt's lymphoma.

Country	ASIR 0-19 year-old	Ratio Child:adult	HDI	sEBV	tEBV
Sulaimani	6.2 (4.7–7.7)	1 : 0.19	0.674	0.92 C	0.83
Egypt	5.2 (4.2–6.2)	1 : 0.05	0.707	Na	0.73
Algeria	1.0 (0.6–1.4)	Na	0.748	0.83 C	0.88
Morocco	5.0 (3.7–6.3)	Na	0.686	Na	Na
Jordan	3.7 (3.1–4.3)	1 : 0.13	0.729	0.98 C	Na
Israel all races	5.0 (4.3–5.7)	Na	0.919	0.87 AYD	0.34
Saudi Arabia	2.8 (2.3–3.3)	Na	0.854	0.98 C	Na
Turkey	3.5 (3.0–4.0)	Na	0.820	0.82 C	0.93
S. Africa all races	1.7 (1.5–1.9)	1 : 2.78	0.709	0.95 EBV 0.78 HIV+	Na
Uganda	21.6 (19.4–23.8)	1 : 0.21	0.544	0.96 EC	>0.95
Cameroon	40.0 (32.9–47.1)	1 : 0.41	0.563	0.97 EC	>0.95
Canada	2.5 (2.3–2.7)	1 : 2.78	0.929	0.95 AYD	Na
US all races	2.4 (2.3–2.5)	1 : 3.85	0.926	0.83 AYD	0.30
UK	2.3 (2.1–2.5)	1 : 4.78	0.932	0.85 AYD	0.10
Germany-West	2.8 (2.4–3.2)	Na	0.947	0.95 AYD	Na
France	4.3 (3.7–4.9)	Na	0.901	0.82 AYD	Na
Italy	4.0 (3.5–4.5)	1 : 0.20	0.892	0.65 AYD	0.62
Spain	4.8 (4.2–5.5)	Na	0.904	0.99 AYD	Na
Japan	1.4 (1.1–1.7)	1 : 2.94	0.919	0.59 AYD	0.29
Korea	2.1 (1.9–2.3)	Ped<Adult	0.916	0.87 AYD	0.00
China	0.4 (0.3–0.5)	1 : 0.55	0.761	0.80 EC	0.32
Thailand	0.8 (0.6–1.0)	Ped>Adult	0.777	0.95 EC	Na
India	0.5 (0.4–0.6)	Ped>Adult	0.645	0.90 EC	Na

Age-specific incidence rates per million 0-19 year old residents are compared together with the ratio of children to adults, Epstein-Barr virus serology, and frequency of tissue EBV latency

*Na* information not available; *ASIR* age-specific incidence rate with 95% confidence interval (95% CI); *HDI* human development index [from the WHO website]; *HIV* human immunodeficiency virus; Ped, pediatric; *EBV* Epstein-Barr virus; *sEBV* population serological EBV frequency; *C* childhood infection, not further specified; *EC* early childhood infection, by 5 years of age; *AYD* adolescent and young adult infection; *tEBV* lymphoma tissue EBER frequency

Europe, the US, and Canada have high developmental indices, and BL is more often an adult rather than a pediatric tumor [4, 5, 14, 27, 28, 32]. The increased frequency of adult BL seems to be determined by EBV exposure being delayed until adolescence and young adulthood. The shift to adult tumors does not seem to abrogate the development of the childhood BL. Despite adult BL becoming more common, Europe, the US, and Canada have rates of pediatric BL that are higher than most of the world, with the highest rates being in Italy, Spain, and France [18].

The Middle East has intermediate developmental indices, and BL is primarily a childhood cancer. In the Middle East, there is a seropositivity rate of more than 90% that seems to be reached at mid-adolescence. The proportion of EBER-positive tumors in Sulaimani, Egypt, Algeria, and Turkey is also high. The exception in the Middle-East seems to be Israel, where data regarding the frequency of adult BL is not reported. One study of Israeli university students indicates EBV seropositivity is frequently negative until adolescence and then increases to 87% by the mid to late twenties [22]. Only 34% of Israeli childhood BL is reported to be EBER positive [21].

In the Far East, Japan and Korea have high development indices, EBV seroconversion often occurs in adolescents and young adults, and adult rates compared to childhood BL are high [35–38]. The BL in these countries is rarely EBER positive [36, 38]. In contrast, China, Thailand, and India have low rates of childhood BL, and adult BL is rare. Development indices are low to intermediate, and EBV infects over 90% of children by five years of age [39–42]. Curiously, however, BL in China seems to rarely be EBER positive [40].

Cameroon and Uganda have the endemic form of BL. The countries have very low development indices, and the populations are exposed to frequent tropical infections including falciparum malaria. Endemic BL does occur in adults but is rare. Cameroon and Uganda contrast with South Africa, where adult BL is nearly 3x more common than pediatric BL [7]. In South Africa, 78% of patients have the combination of HIV and EBV infections, but rates of pediatric BL are markedly lower than Equatorial African and approximately half the rates of the US [6, 7]. The lower South African rates compared to the US seems to reflect the predominantly black population of South Africa and the mostly white population of the US.

### EBV serology in Sulaimani outpatients

Serum specimens of 515 patients were tested (Table 4). The instrument recorded 423 IgG anti-VCA (82.1%) and 14 IgM anti-VCA (2.7%) results as positive. All positive IgM results except one were positive for IgG. The IgM positive and IgG negative patient was 13-years-old and likely a primary infection but was otherwise not identifiable. The pattern of EBV infection was unimodal. At one year of age, 47% of infants were IgG positive, and at four years, 63% of children were positive. Seropositivity was 88% at 14, and after 14 years of age, a plateau was reached. For adults 20-69 years old, 135 of 146 specimens were seropositive, a rate of 92%.

**Table 4:** Serological testing among 515 non-oncology outpatients for Ig Epstein-Barr virus viral capsid antigen

Age (years)	No. tested	No. positive	Fraction positive
0.25-0.99*	20	9	0.45
1	34	16	0.47
2	33	17	0.52
3	19	13	0.68
4	27	17	0.63
5	25	21	0.84
6	35	24	0.69
7	30	23	0.77
8	26	20	0.77
9	13	9	0.69
10–14	74	65	0.88
15–19	33	32	0.97
20–24	22	16	0.73
25–29	22	22	1.00
30–34	15	14	0.93
35–39	19	17	0.89
40–49	29	29	1.00
50–59	25	23	0.92
60–69	14	14	1.00
Total	515	401	0.78

Results are tabulated by age and as a total.

*Under one year of age, subjects less than three months old were not reported, because maternal antibodies produced passive IgG positive and IgM negative reactions. Three such subjects were found at three and 12 days and two months.

## Discussion

Outside of the very high incidence areas endemic to Equatorial Africa, there is striking variation in the frequency of BL that seems to be related to the age of EBV exposure. BL is rare in China, Thailand, and India, where nearly all children are infected by EBV before five years of age. In the Middle East, BL has incidence rates that are well below Equatorial Africa but much higher than any country in Asia. In the Middle east, the childhood BL has a high frequency of EBER positivity, and few tumors are reported in adults [8–10, 19, 21]. Data from Sulaimani, Qatar, and Bahrain indicate that nearly half of EBV seroconversion in the Middle East occurs between four and 15 years old [15–17]. In North America and Northern Europe, childhood rates of BL are somewhat lower than the Middle East, but BL is more common in adults than children [4, 5, 27, 32, 36, 37]. In these countries, much of the EBV seroconversion is in adolescents and young adults, and the frequency of EBER-positive tumors is low.

The variations in regional EBV exposure and evidence for tumor EBV latency confuse our understanding of the relationship between EBV and BL. EBV is an upper respiratory virus that enters the nasopharynx and infects epithelium and lymphocytes, with B cells being the primary target [45].

The early EBV infection stimulates lymphoblastic proliferation. The lymphoblasts enter the germinal centers and undergo somatic hypermutations in heavy and light chain genes [2, 45]. Random translocations between the chromosome 8 MYC oncogene and the chromosome 14 heavy chain or an alternative light chain locus complicate these hypermutations, with the MYC translocation being considered an essential carcinogenic event [2, 45]. The EBV-stimulated lymphoblastic proliferation usually becomes quiescent, and approximately 2% of cells enter a type 3 latency as memory B cells. In a type 3 latency, cells produce nuclear EBER but have little expression of other viral components, and viral replication is limited [2, 45, 46]. If infected memory B cells encounter their cognate antigen, they can transform into proliferating mature B cells, and if they bear the MYC translocation, they can become BL [2, 45].

The age of exposure to EBV affects latency and the risk of tumor development, with the early childhood exposure in much of the world being associated with a low risk of BL [2]. The endemic BL is an exception, and the very high risk of lymphoma is attributed to coexisting malaria that stimulates B cells. This stimulation augments the frequency of somatic hypermutations and MYC translocations, with higher translocation rates increasing the risk of BL [2, 45].

Even in countries with a late age of EBV infection, overall seropositivity is at least 70-80%. The nearly universal nature of the infection and variable BL rates outside of endemic regions questions the role of EBV in the development of the tumor. The relationship of BL to germinal center MYC translocations means that virtually any antigenic stimulus could promote tumor development [2, 45].

While pediatric BL has a generally uniform histologic and clinical appearance, BL in adults can present challenges that may not be adequately addressed in current Middle East pathology practices. The WHO classification of lymphoid neoplasms was introduced into the region around 2010, and the complexity of the classification can be difficult for a general pathologist anywhere [9]. BL is defined as a high proliferation rate mature B-cell lymphoma with an isolated MYC translocation [2, 5]. The distinction between BL and diffuse large B-cell lymphoma can be a problem in children and adults, and high-grade B-cell lymphomas of adults histologically and clinically overlap with BL [2, 5, 47]. To make matters even more complex, data on molecular profiling indicate that BL may not always have a MYC abnormality and that the germinal center CD10 and BCL6 positive phenotype, by itself, might be sufficient for the diagnosis [47].

Analyzing international data provokes as many questions as it provides answers. Detailed information is available through IICC-3 on virtually all pediatric cancers [18]. For adults, the WHO Globocan website records international data for non-Hodgkin lymphoma, but there is no separation into subtypes [48]. This is a problem for BL. Most adult BL occurs in immunocompetent patients, and increased adult BL rates may be related to the aging of populations having late exposure to EBV [4]. But in Europe, North America, Japan, and Korea, it is notable that the higher frequency of BL in adults is associated with an accompanying change to EBER-negative BL in children, and in Europe and North America, the rates of childhood BL remain elevated.

BL is an important adult tumor, having a frequency approaching mantle cell lymphoma [5]. The absence of details in Globocan requires investigators to depend upon regional publications. US studies using the SEER database are considered representative of the general population [4]. In many other countries, the studies are hospital-based and may not embody the entire nation.

Recent investigations of BL emphasize molecular mechanisms related to MYC activity and rarely include an analysis of EBV latency [47]. There also seems to be a declining interest in the relationship between the spread of EBV and lymphoma, although, outside of endemic regions, high rates of early childhood EBV infection are associated with low rates of EBV-positive BL [2–4]. When infections are more frequent in adolescents and young adults, rates of BL are moderately high, and the frequency of tumor EBV latency is low. These are clear relationships indicating that alternate pathogenetic mechanisms not involving EBV latency or MYC mutations can promote the BL phenotype.

In Sulaimani, several childhood and adult EBV-related tumors are recognized. These include nasopharyngeal carcinoma, NK/T-cell lymphoma of nasal type, and EBV-related large B cell lymphoma. As a group, they comprise less than 0.1% of our cancers. EBV-positive post-transplant lymphoproliferative disease occurs in approximately 1% of our kidney transplants, a frequency similar to the US [49, 50]. All of these tumors are occurring in a population that has a high EBV seroconversion rate that reaches its peak at about 15 years of age.

BL in the Middle East has an incidence that resembles countries with high indices of development. We believe this BL is a sporadic disease, with EBV being the only identifiable promoting factor. Most of the Middle East, including Iraq, has been malaria-free since the late 1990s, and non-Hodgkin lymphomas associated with other infections such as human herpes virus-8, *Helicobacter pylori,* and hepatitis B and C are uncommon [9, 51].

The reason that adult BL is rare in the Middle East is uncertain. If data from Sulaimani, Bahrain, and Qatar are representative of the Mid East, it may be related to a high rate of childhood EBV exposure but with a delayed plateau until mid adolescence [15–17]. This seems to be a third pattern of EBV infection between the early childhood exposure of countries with low developmental indices and the adolescent and young adult exposure of countries with high socioeconomic development. It is notable that until adulthood, rates of Middle Eastern BL resemble those of Southern Europe more than any other region of the world.

The determination of the Sulaimani EBV seropositivity used de-identified clinical specimens initially obtained for other diagnostic purposes. This is a limitation to any generalization about infection rates in the region. Nevertheless, the limitation is shared with other major studies of population EBV exposure that also analyzed stored clinical specimens or blood donations [12, 15, 16, 22, 26, 29–32, 46]. The social strata of patients seeking medical attention may be different than the general population, and socio-economic factors can influence infection [12, 14]. A study of EBV infection in a general population would raise issues of harm and confidentiality and be difficult to design short of a large project using stored specimens as a general health survey [14]. This leaves the analysis of previously clinical specimens the most available, although less than ideal, method for inferring viral exposure.

## Conclusion

Sulaimania and most of the Middle East have a unique frequency of BL. It has an incidence among children that is much lower than the endemic BL of Equatorial Africa but is high compared to the sporadic BL of most of the world. These findings are relevant to a regional pattern of Middle Eastern BL that may be transitioning to even higher rates, particularly among adults. The suspected transition may be related to a shift in the age of EBV exposure from early childhood to early adolescence.

## Data Availability

Compiled data and calculations are stored in Excel files in the Shorsh Hospital Pathology Department and will be made available upon request to the corresponding author, MDH.

## Abbreviations

BL: Burkitt’s lymphoma
WHO: World Health Organization
IICC-3: International Incidence of Childhood Cancer-3
EBV: Epstein-Barr virus
HIV: Human immunodeficiency virus
EBER: EBV-encoded small non-polyadenylated mRNA
FNA: Fine needle aspiration
VCA: Viral capsid antigen
ASIR: Age-standardized incidence rate
95% CI: 95% confidence intervals
